# Efficacy of a third-generation oncolytic herpes simplex virus in refractory soft tissue sarcoma xenograft models

**DOI:** 10.1016/j.omto.2022.04.010

**Published:** 2022-04-26

**Authors:** Masahiko Hatta, Masaki Kaibori, Hideyuki Matsushima, Terufumi Yoshida, Tadayoshi Okumura, Mikio Hayashi, Kengo Yoshii, Tomoki Todo, Mitsugu Sekimoto

**Affiliations:** 1 Department of Surgery, Kansai Medical University, 2-5-1 Shinmachi, Hirakata, Osaka 573-1191, Japan; 2 Research Organization of Science and Technology, Ritsumeikan University, Kusatsu, Shiga, Japan; 3 Department of Cell Physiology, Institute of Biomedical Science, Kansai Medical University, Hirakata, Japan; 4 Department of Medical Statistics, Kyoto Prefectural University of Medicine, Kyoto, Japan; 5 Division of Innovative Cancer Therapy, Advanced Clinical Research Center, Institute of Medical Science, The University of Tokyo, Minato-ku, Tokyo, Japan

**Keywords:** oncolytic virus, herpes simplex virus, T-01, malignant soft tissue tumor, mouse xenograft model

## Abstract

Malignant soft tissue tumors, particularly highly malignant leiomyosarcomas, are resistant to chemotherapy and associated with a poor prognosis. T-01, a third-generation genetically modified herpes simplex virus type 1, replicates in tumor cells alone and exerts a cell-killing effect. The current study aimed to investigate the antitumor effect of T-01, which is a novel treatment for leiomyosarcoma. *In vitro*, six human cell lines and one mouse sarcoma cell line were assessed for T-01 cytotoxicity. *In vivo*, the efficacy of T-01 was examined in subcutaneously transplanted leiomyosarcoma (SK-LMS-1) cells and subcutaneously or intraperitoneally transplanted mouse sarcoma (CCRF S-180II) cells. Cytokines were assessed using ELISpot assay with splenocytes from the allogeneic models for immunological evaluation. T-01 showed cytotoxicity in all seven cell lines (p < 0.001). In the SK-LMS-1 xenotransplantation model, tumor growth was suppressed by T-01 administration (p = 0.02). In the CCRF S-180II subcutaneous tumor model, bilateral tumor growth was significantly suppressed in the T-01-treated group compared with the control group (p < 0.001). In the peritoneal dissemination model, T-01 treatment caused significant survival prolongation compared with the control (p < 0.01). In conclusion, third-generation genetically modified herpes simplex virus type 1 may be an effective novel therapy against refractory sarcomas.

## Graphical abstract

**Figure:**
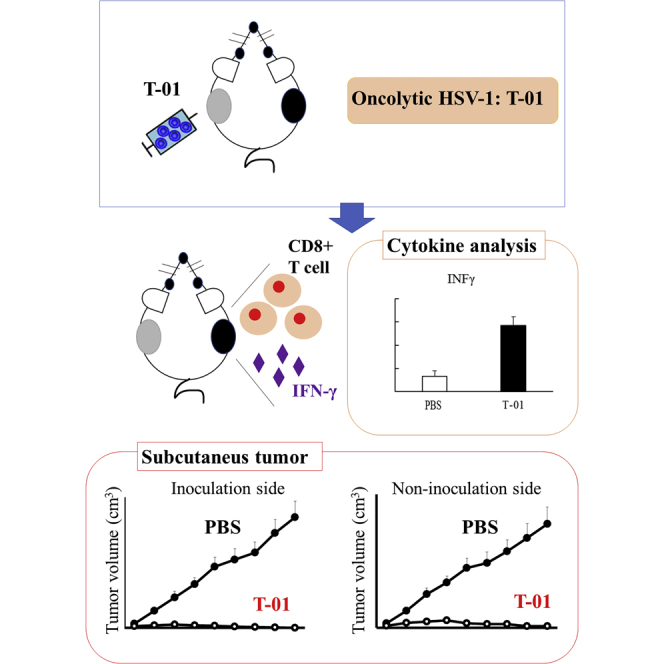

## Teaser

T-01, a third-generation genetically modified herpes simplex virus type 1, can effectively inhibit tumor growth in the mouse models of rhabdomyosarcoma and leiomyosarcoma. Oncolytic viral therapy using third-generation oHSV may be a novel treatment for refractory sarcoma.

## Introduction

Soft tissue tumors generally refer to tumors that arise from or differentiate into non-epithelial tissues such as muscular, adipose, fibrous, vascular, and peripheral nerve tissues. There are more than 100 types of benign and malignant soft tissue tumors, including approximately 40 types of malignant soft tissue tumors.^1^ The main histological types of malignant soft tissue tumors are liposarcoma, undifferentiated sarcoma, rhabdomyosarcoma (RMS), leiomyosarcoma (LMS), synovial sarcoma, and malignant peripheral nerve sheath tumor.^2^ Due to the extremely poor outcome in patients with LMS and RMS, innovative treatment strategies are required.^3^, ^4^, ^5^, ^6^ Of these, advanced-stage LMS is associated with a poor prognosis and is not responsive to second-line chemotherapy.^3^^,^^4^ Surgery is a curative treatment option for localized LMS, regardless of the site of origin. However, in LMS originating from the retroperitoneum or abdominal cavity or in cases involving a tumor with a diameter exceeding 10 cm, an adequate resection margin is challenging to secure with surgical treatment, and the prognosis is often poor.^3^^,^^4^ There are several cases of advanced-stage uterine LMS with metastasis to the lymph nodes, hematogenous metastasis to the lungs and liver, and peritoneal dissemination.^6^ The incidence of stage IV LMS is higher than that of stage I LMS. Thus, uterine LMS is characterized by distant metastasis.^7^ The 5-year survival rates of patients with uterine LMS according to the Federation of Gynecology and Obstetrics 2008 staging system are 55.4% for stage I, 32.6% for stage II, 24.6% for stage III, and 13.1% for stage IV.^8^, ^9^, ^10^, ^11^ To date, there is no effective chemotherapy for uterine LMS. However, doxorubicin-based therapy is recommended as the first-line treatment for locally advanced or metastatic LMS arising outside the uterus.^12^ However, no second-line treatment has been established for cases in which doxorubicin-based therapy is not effective. A combination of surgery, radiation therapy, and chemotherapy is the standard of care for RMS. In the RMS study groups in Europe, the United States, and Japan, the 3-year progression-free survival rates were 80% to 100% for individuals at low risk of developing RMS, 50% to 80% in those at intermediate risk, and 30% to 50% in those at high risk.^13^ Currently, approximately 70% of pediatric patients with non-metastatic RMS are cured with multidisciplinary treatment combining radiotherapy and chemotherapy.^14^ However, these treatments have not improved the RMS cure rate in adults, and the prognosis of RMS remains extremely poor (with an overall survival rate of only 20%–40%).^15^

Herpes simplex virus type 1 (HSV-1) infects different varieties of cell types and exhibits strong cytotoxicity, thereby making it an attractive treatment for sarcoma. HSV-1 may be suitable for clinical application because it is not affected by circulating antibodies in cell-to-cell transmission.^16^ Hence, oncolytic viral therapy can specifically destroy tumor cells because mutations in genes correlated with viral DNA synthesis, viral virulence, or both can promote viral replication in cancer cells. Oncolytic HSV-1 (oHSV-1) can be a novel treatment for malignant tumors because it induces selective replication and damage in tumor cells. Furthermore, it is not associated with cross-resistance to other therapeutic strategies such as chemotherapy.^17^ oHSV is undergoing phase I–III clinical trials for the treatment of solid cancers.^18^, ^19^, ^20^, ^21^, ^22^

The oHSV G207 variant from HSV-1 strain F has deletions in both copies of the γ34.5 gene and a LacZ insertion within the ICP6 gene, thereby inactivating ICP6. This phenomenon allows viral replication in cancer cells to compensate for these mutations, but not in normal cells, including neurons.^23^ In clinical applications, the LacZ gene is useful as a viral replication marker. The mutant G47Δ is derived from the G207 variant by introducing a third deletion in the α47 gene that overlaps with the US11 promoter.^24^ The expression of the US11 gene is accelerated, and it functions as a second site suppressor of the γ34.5 mutation to restore virus replication ability, which is weakened in the γ34.5 deletion HSV-1 in tumor cells alone.^24^ Compared with G207, G47Δ can more efficiently replicate and induce the presentation of major histocompatibility complex (MHC) class I molecules while maintaining the safety profile of G207.^24^ These properties enhance the response of cytotoxic lymphocytes to tumor cells and improve the therapeutic efficacy of the virus, as shown in animal models of brain tumors, prostate and breast cancer, and neurofibroma.^24^, ^25^, ^26^, ^27^ Clinical trials about G47Δ in patients with recurrent glioblastoma, olfactory neuroblastoma, or prostate cancer are under way in Japan.^28^

T-01, a third-generation genetically modified HSV-1, has a genomic structure similar to that of G47Δ.^29^ Recent studies have shown that T-01 effectively inhibits the growth of human hepatocellular carcinoma and neuroendocrine tumors in mice.^30^^,^^31^ In human hepatocellular carcinoma, tumor inhibition of tumor is attributed to the immune activity of T-01. The current study aimed to examine the antitumor activity of T-01 in LMS and RMS cell lines and in mouse xenograft models. Moreover, the immune response of the host in a mouse allogeneic transplant model using a mouse sarcoma cell line was evaluated.

## Results

### Cytopathic effects of T-01 and virus yields *in vitro*

**Figure 1:**
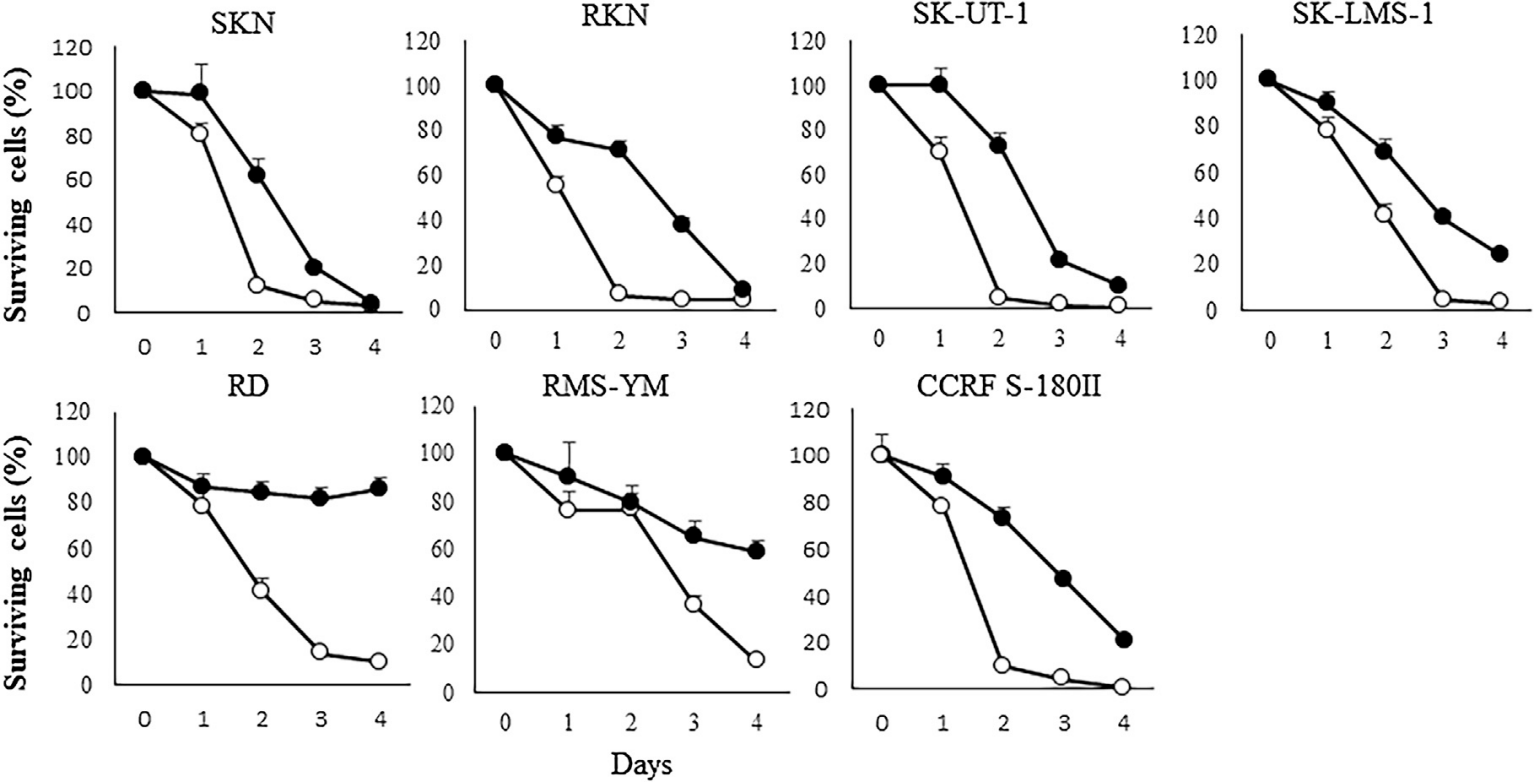
Cytotoxic activity of T-01 *in vitro* Cell lines (such as SKN, RKN, SK-UT-1, SK-LMS-1, RD, RMS-YM, and CCRF S-180II) were treated with the T-01 virus (MOI = 0.01 [filled circles] or 0.1 [open circles]) and were incubated for a specific number of days. The number of surviving cells was counted and expressed as a percentage relative to that in the PBS control at each time point. Data are expressed as mean ± SE (n = 6/time point).

The effects of original T-01^29^ on human LMS (such as SKN, RKN, SK-UT-1, and SK-LMS-1), human RMS (RD and RMS-YM), and mouse sarcoma (CCRF S-180II) cells were examined using an *in vitro* cytotoxicity assay (n = 6 per group). T-01 infection inhibited cell proliferation on days 1 to 4 (Figure 1). When infected at a low MOI (0.01), on day 4, the populations of SKN, RKN, SK-UT-1, and SK-LMS-1 cells were reduced to <10%, <10%, <20%, and <30%, respectively. The CCRF S-180II cell population was reduced to <30% and the RMS-YM and RD cell populations to <60% and <90%, respectively, compared with the control (PBS). There was a tendency for growth inhibition in RMS-YM and RD cells after infection with T-01. When infected at a high MOI (0.1), the proportions of all cell lines (SKN, RKN, SK-UT-1, SK-LMS-1, RD, RMS-YM, and CCRF S-180II) were significantly reduced on day 4 to <20% in RMS-YM cells (p < 0.001) and <10% in other cell lines (p < 0. 001), compared with the control (PBS).

**Figure 2:**
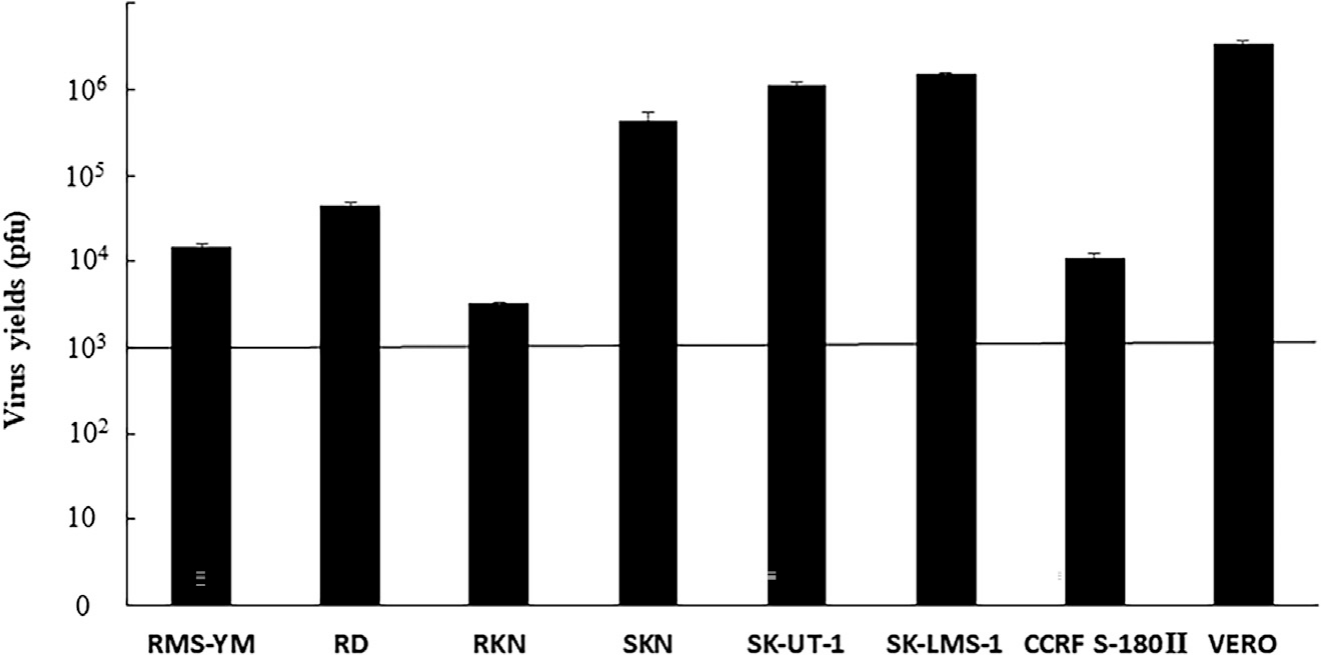
Viral replication of T-01 *in vitro* *In vitro* virus yields were determined using plaque assays 48 h after infection with T-01 (MOI = 0.01) in vero or sarcoma cells (such as SKN, RKN, SK-UT-1, SK-LMS-1, RD, RMS-YM, and CCRF S-180II) (5 × 10^5^ cells/well). The bold line indicates the initial virus concentration. Data are expressed as mean ± SE (n = 6).

To examine viral replication at a low MOI (0.01), the cells were first infected with the virus at a concentration of 5.0 × 10^3^ plaque-forming units (PFU) and were cultured for 48 h. Then, the *in vitro* virus yield was measured using the plaque assay. Only RKN cells had a 0.64-fold decrease in virus yield. Meanwhile, other cell lines, specifically SKN, SK-UT-1, SK-LMS-1, RD, RMS-YM, and CCRF S-180II, had 123-, 234-, 302-, 9.2-, 3.0-, and 2.3-fold increase in the yield, respectively (Figure 2). T-01 showed good replication ability in all cultured cell lines except for RKN cells.

### Effects of T-01 in mice with subcutaneous tumors

**Figure 3:**
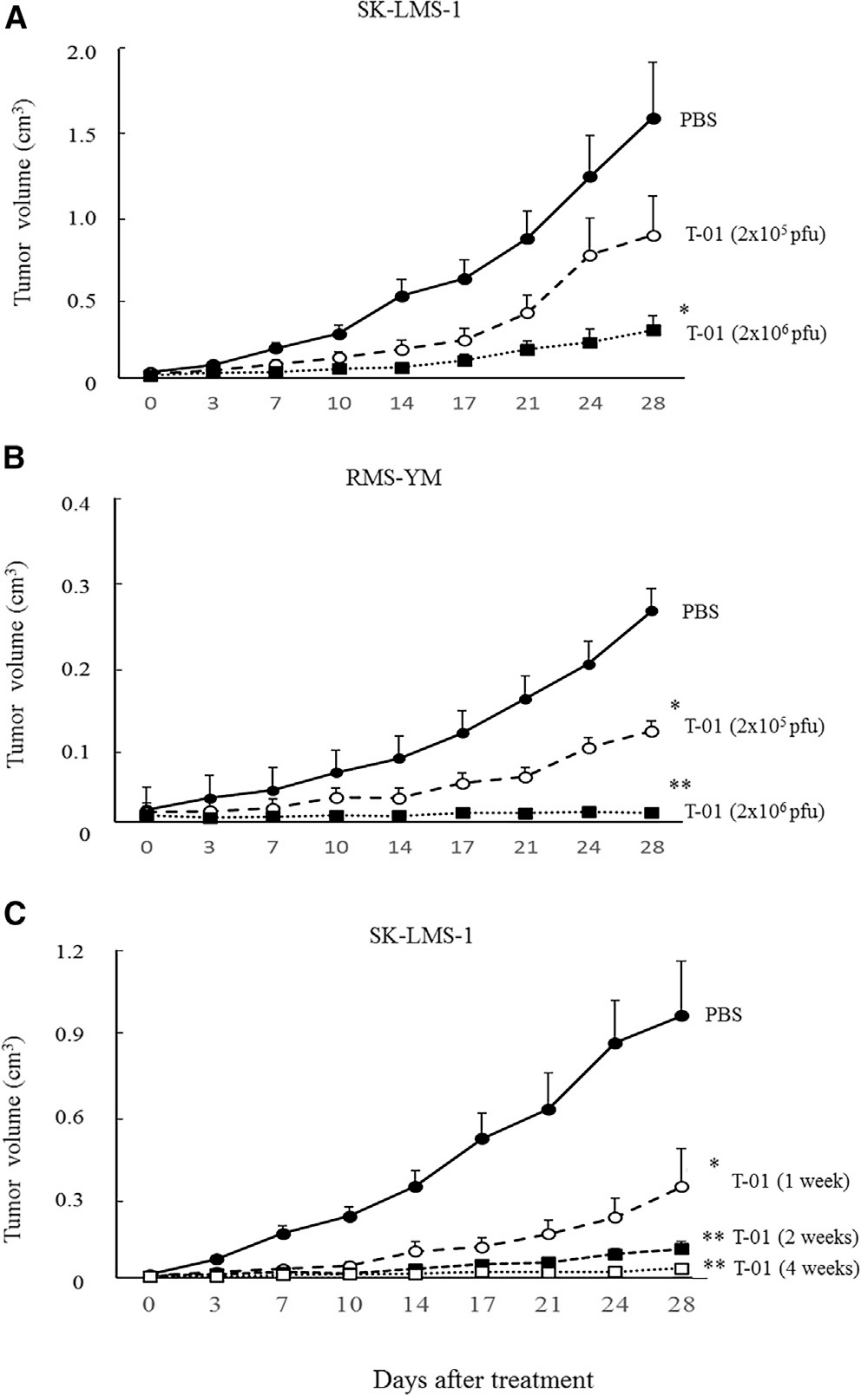
Dose-dependent antitumor effect of T-01 using different administration protocols in mice with subcutaneous tumors (A) SK-LMS-1 and (B) RMS-YM cells were implanted subcutaneously in female athymic mice. Tumors were inoculated twice weekly (days 0 and 3) with T-01 (2.0 × 10^5^ PFU [open circle] or 2.0 × 10^6^ PFU [filled squares]) or PBS (solid circles). Data are expressed as mean ± SE (n = 10 mice/group). (C) SK-LMS-1 cell-derived tumors in female athymic mice were inoculated twice weekly (days 0 and 3) with PBS (filled circles) or T-01 (2.0 × 10^6^ PFU) at various time points: 2 (open circle), 4 (filled squares), or 8 (open squares) inoculations. Data are expressed as mean ± SE (n = 10 mice/group). ∗p < 0.05 and ∗∗p < 0.01 versus PBS treatment.

The effect of T-01 was examined in athymic mice harboring subcutaneous tumors derived from human LMS (SK-LMS-1) or human RMS (RMS-YM) cells. Three treatment groups were evaluated: PBS, 2.0 × 10^5^ PFU T-01, and 2.0 × 10^6^ PFU T-01 (n = 10 per group). Tumor growth, as measured using tumor volume, was more likely to be slower in mice inoculated with 2.0 × 10^6^ compared with those inoculated with 2.0 × 10^5^ T-01. Moreover, it was inhibited in the T-01 group in a dose-dependent manner compared with the PBS group (Figures 3A and 3B). Next, we examined tumor growth in response to different T-01 dosing protocols. In this experiment, athymic mice with subcutaneous tumors generated from SK-LMS-1 cells (human LMS cell line) were infected with PBS or T-01 twice a week for 1, 2, or 4 weeks (n = 8 per group) at a constant virus concentration of 2.0 × 10^6^ PFU. The tumor volume was more likely to be smaller in the 2- and 4-week inoculation groups than in the 1-week inoculation group. All T-01 treatment groups had significant tumor growth inhibition compared with the PBS group on day 28 (PBS: tumor volume ±SE, 0.959 ± 0.201 cm^3^; T-01 at 1 week: 0.333 ± 0.141, p = 0.003; T-01 at 2 weeks, 0.105 ± 0.027, p < 0.001; T-01 at 4 weeks, 0.034 ± 0.018, p < 0.001) (Figure 3C). Therefore, the antitumor effect of T-01 was dependent on the concentration and frequency of administration.

### Histological examination

**Figure 4:**
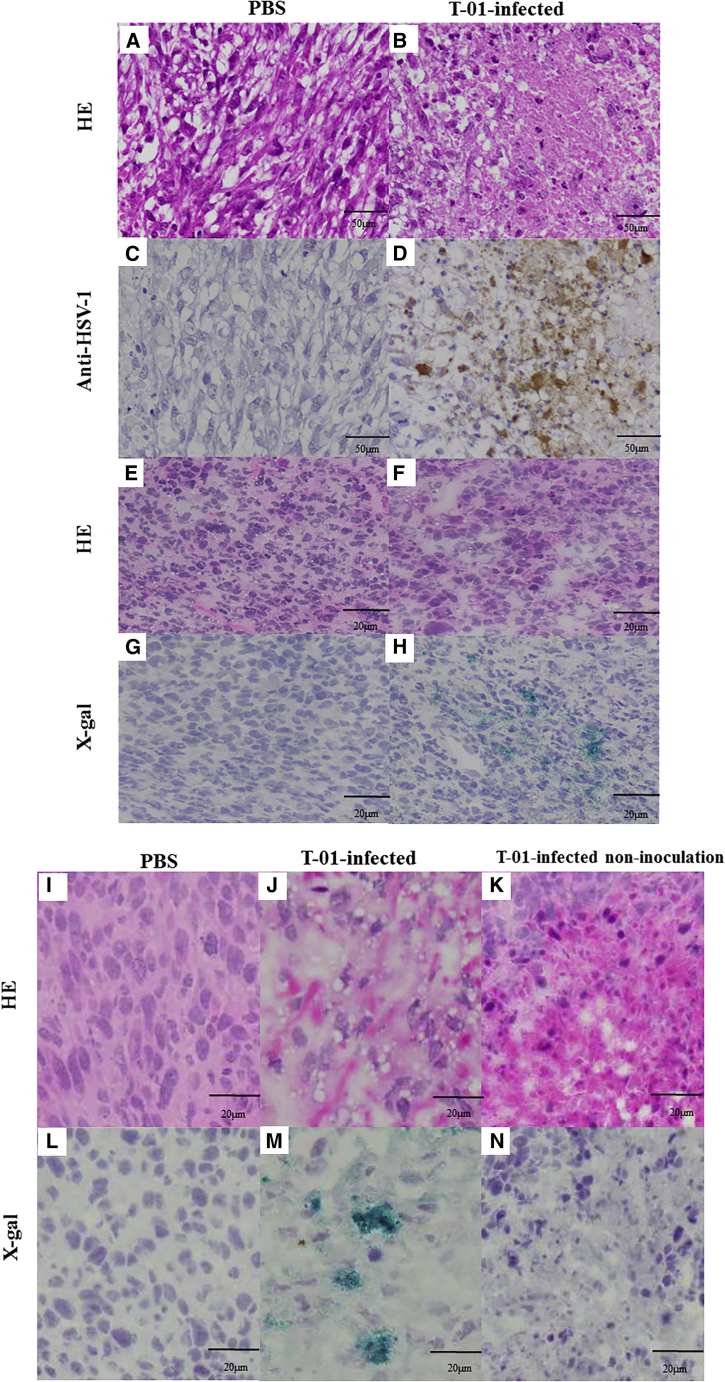
Immunohistochemical analysis of HSV-1 in xenograft tumors Athymic mice were subcutaneously transplanted with SK-LMS-1 (A–D) and RMS-YM (E–H) cells, and the resulting subcutaneous tumors were inoculated with T-01 (2.0 × 10^6^ PFU) or PBS twice weekly (days 0 and 3). Mice were euthanized on day 7 after inoculation, and tissue sections were stained with H&E (A, B, E, and F), anti-HSV-1 antibody (C, D), or X-gal (G, H). ICR mice with bilateral subcutaneous tumors arising from the CCRF S-180II cells (I–N) were established, and one of the bilateral subcutaneous tumors was inoculated with T-01 (2.0 × 10^6^ PFU) or PBS twice weekly (days 0 and 3). Mice were euthanized 7 days after inoculation, and histological images of tissue sections stained with H&E (I–K) or X-gal (L–N) are shown. Representative images from these experiments are presented.

After 1 week of T-01 inoculation, tumors derived from the human LMS cell line SK-LMS-1 and the human RMS cell line RMS-YM were positive for HSV-1 and 5-Bromo-4-chloro-3-indolyl-β-D-galactopyranoside (X-gal) staining (Figures 4D and 4H), respectively. H&E staining showed that tumor cells were destroyed at the site of virus amplification (Figures 4B and 4F). By contrast, in mice inoculated with PBS for 1 week, HSV-1 positivity was not observed in the tumors (Figures 4C and 4G). H&E staining indicated no destruction in tumor cells (Figures 4A and 4E). However, tumors derived from the mouse sarcoma cell line CCRF S-180II and treated directly with T-01 were positive for X-gal (Figure 4M). H&E staining showed tumor cell destruction at the site of virus amplification in immunocompetent mice (Figure 4J). Tumors on the non-inoculated side of mice treated with T-01 were not positive for X-gal (Figure 4N). However, H&E staining showed destruction of tumor cells (Figure 4K). In mice inoculated with PBS for 1 week, there was no X-gal positivity (Figure 4I), and H&E staining revealed no destruction of tumor cells (Figure 4L).

### Efficacy of T-01 treatment in immunocompetent mice with bilateral subcutaneous tumors derived from CCRF S-180II cells

**Figure 5:**
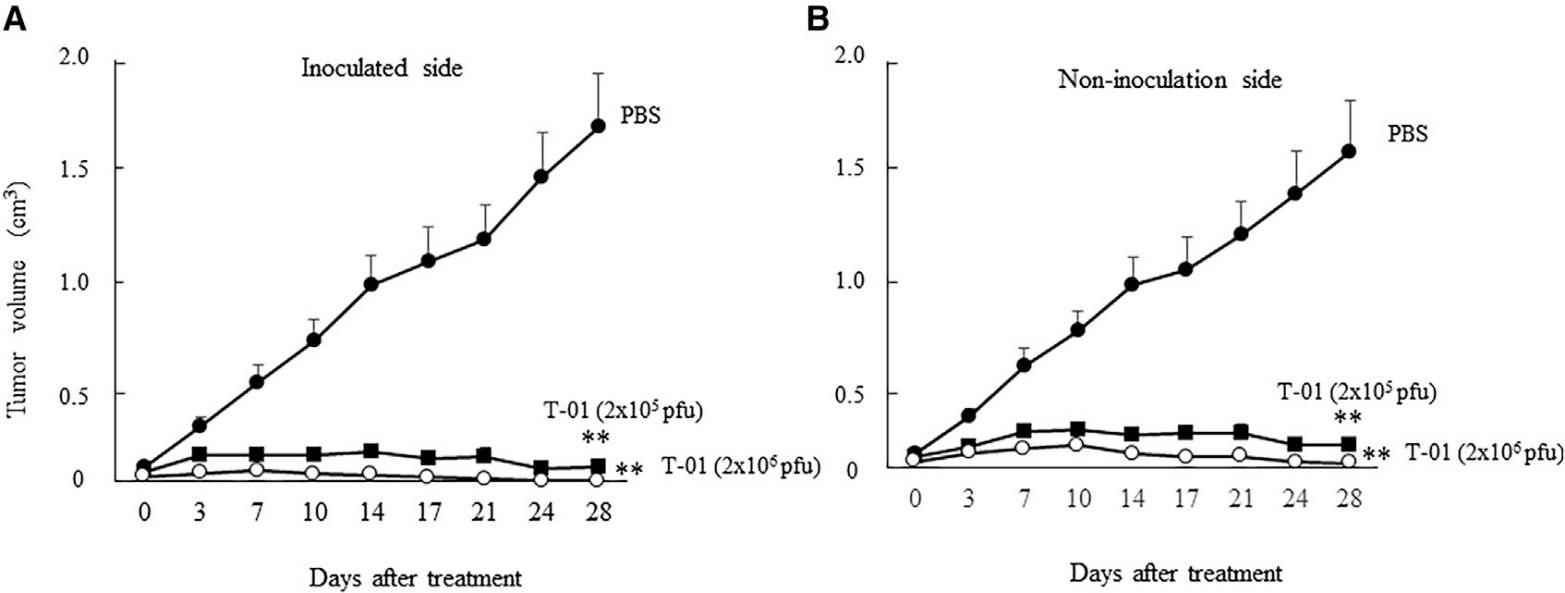
Cytopathic effects of T-01 in immunocompetent mice with bilateral subcutaneous tumors Male ICR mice harboring subcutaneous CCRF S-180II tumors in their bilateral flanks were established. Tumors on one side were inoculated with T-01 (2.0 × 10^5^ PFU [solid square] or 2.0 × 10^6^ PFU [open circle]) or PBS (solid circle) twice weekly (days 0 and 3). Tumor volumes on the (A) inoculated and (B) uninoculated sides were evaluated. Data are presented as mean ± SE (n = 8 mice/group). ∗∗p < 0.01 versus PBS treatment.

ICR mice with bilateral subcutaneous tumors derived from CCRF S-180II cells on their backs were inoculated with T-01 (at 2.0 × 10^5^ or 2.0 × 10^6^ PFU) on one side only. Tumor growth was compared with respect to treatment (PBS and 2.0 × 10^5^ or 2.0 × 10^6^ PFU T-01) and the inoculated versus non-inoculated side (n = 8 per group). Both the T-01-inoculated and non-inoculated sides had significantly suppressed tumor growth compared with the PBS group on day 28 (p < 0.001; Figure 5). Mice inoculated with 2.0 × 10^6^ PFU T-01 had a smaller tumor volume than those inoculated with 2.0 × 10^5^ PFU T-01. Tumor growth was suppressed in a dose-dependent manner in the T-01 group compared with the PBS group.

### Induction of cytokines in mice transplanted with CCRF S-180II cells

**Figure 6:**
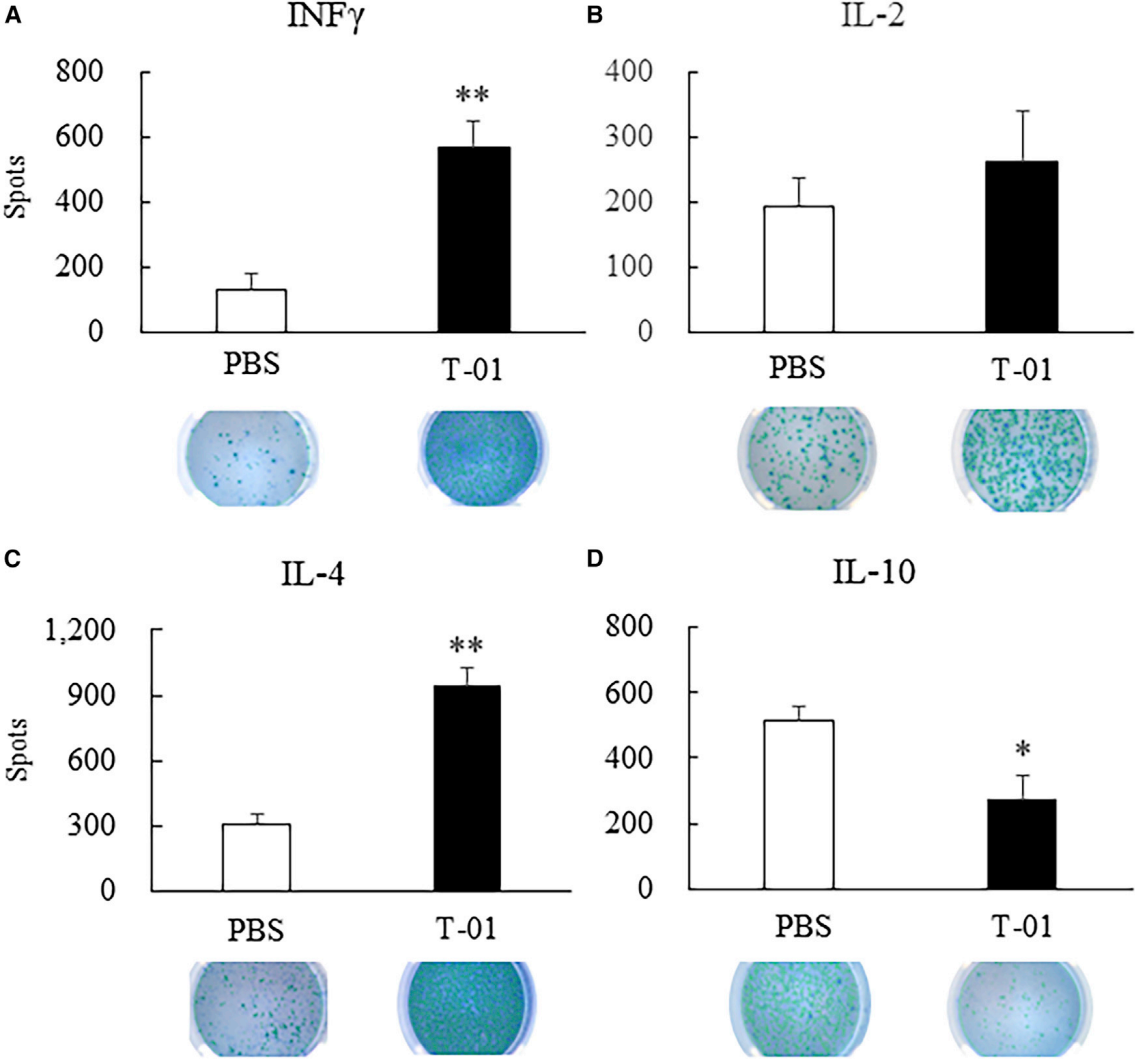
IFNγ, IL-2, IL-4, and IL-10 levels in the splenocytes of CCRF S-180II tumor-bearing mice Male ICR mice with subcutaneously established CCRF S-180II tumors on the bilateral dorsum were treated with PBS or T-01 (2.0 × 10^6^ PFU) twice weekly (days 0 and 3). ELISPOT assays for (A) IFNγ, (B) IL-2, (C) IL-4, and (D) IL-10 were performed with the splenocytes of each group. Data are presented as mean ± SE (n = 5 mice/group). ∗p < 0.05 and ∗∗p < 0.01 versus PBS treatment.

ICR mice with bilateral back subcutaneous tumors derived from the mouse sarcoma cell line CCRF S-180II were inoculated with T-01 on one side only. Mice inoculated with 2.0 × 10^6^ PFU T-01 (n = 5) secreted significantly higher levels of interferon gamma (IFN-γ) and interleukin (IL)-4 than PBS-treated mice (n = 5; p < 0.01) (Figures 6A and 6C). A higher level of IL-2, but a lower level of IL-10, was secreted in the T-01 group than in the PBS group (p < 0.05) (Figures 6B and 6D).

### Immunological analysis of CD4+ and CD8+ lymphocytes

ICR mice with bilateral subcutaneous tumors derived from CCRF S-180II mouse sarcoma cells on their backs were inoculated with T-01 (2.0 × 10^6^ PFU) on one side only. CD4+ and CD8+ lymphocytes were counted and compared among PBS-treated mice and the inoculated and non-inoculated sides of T-01-treated mice (n = 3 per group). The proportions of CD4+ and CD8+ lymphocytes significantly increased in the T-01 inoculation group compared with the PBS group (p < 0.01; Figure S1). The proportions of CD4+ and CD8+ lymphocytes were more likely to be higher on the T-01 non-inoculated side of T-01-treated mice than in PBS-treated mice (p = 0.489 and 0.0718, respectively; Figure S1).

Next, the number of CD4+ and CD8+ lymphocytes in the spleen of T-01-treated mice was examined. ICR mice with bilateral subcutaneous tumors derived from CCRF S-180II mouse sarcoma cells on their backs were inoculated with T-01 (2.0 × 10^6^ PFU) on one side only. The number of CD4+ and CD8+ lymphocytes in the spleen was assessed and compared between the PBS- and T-01-treated mice (n = 4 per group). Mice treated with T-01 had a significantly higher number of CD4+ and CD8+ lymphocytes than those treated with PBS (p = 0.026 and 0.018, respectively; Figure S2).

### Efficacy of T-01 in peritoneal dissemination models

**Figure 7:**
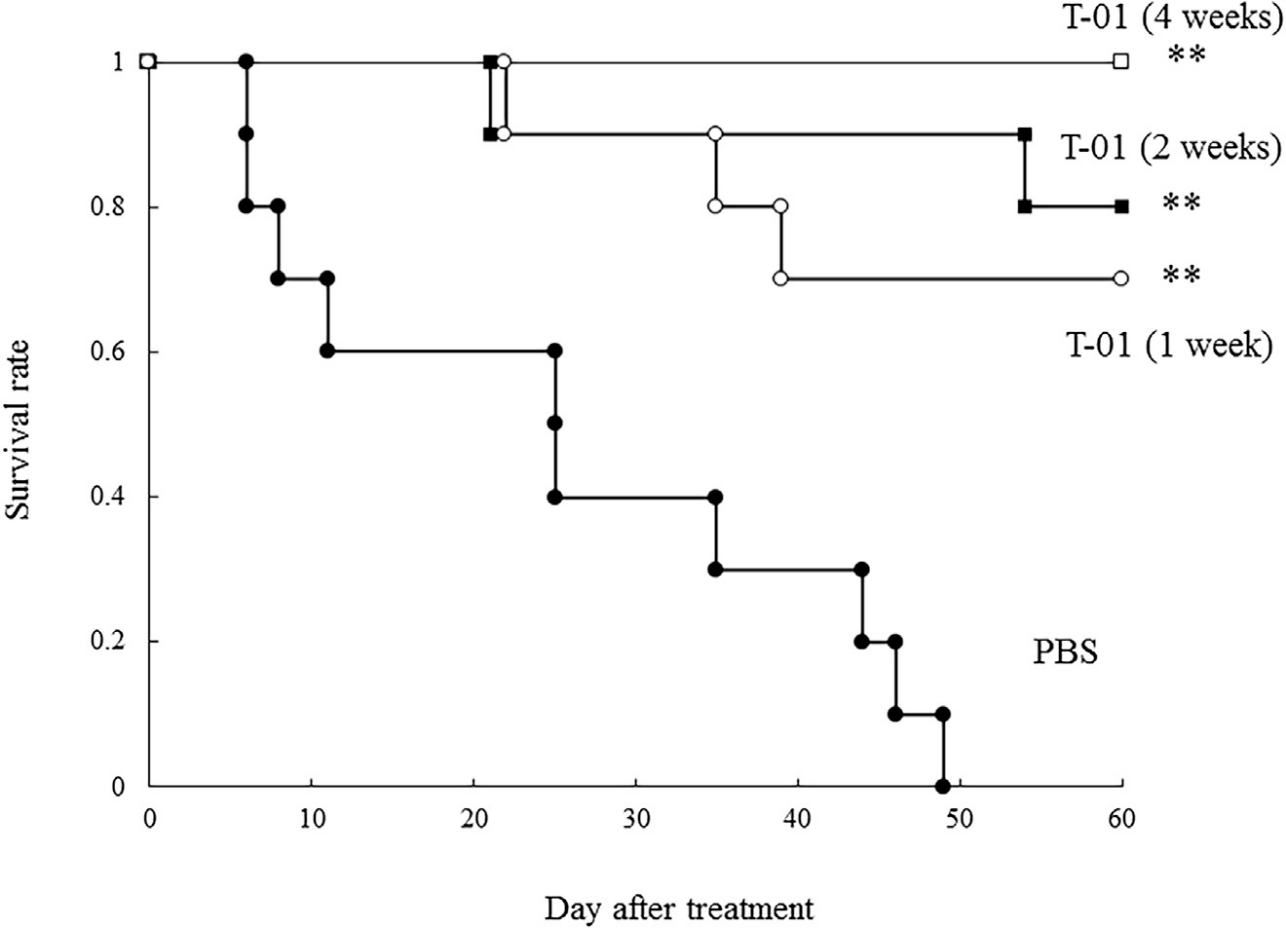
Cytotoxic effect of T-01 in mice with peritoneal dissemination Mouse cells (CCRF S-180II) were transplanted into the peritoneal cavity of male ICR mice. The graph shows the survival rate of CCRF S-180II tumors after intraperitoneal inoculation of PBS (filled circles) or T-01 (2.0 × 10^6^ PFU) twice weekly (days 0 and 3) at various time points (1 week [open circles], 2 weeks [filled squares], or 4 weeks [open squares]). Data are presented as mean ± SE (n = 10 mice/group). ∗∗p < 0.01 versus PBS treatment.

Peritoneal metastatic tumors were established in ICR mice implanted with CCRF S-180II cells. Mice were then inoculated intraperitoneally with PBS or T-01 (2.0 × 10^6^) twice a week, and survival was examined. The mice were infected with PBS or T-01 twice a week for 1, 2, and 4 weeks (n = 10 per group). In the PBS group treated with CCRF S-180II cells, several gross peritoneal tumor nodules were formed. Meanwhile, in the T-01 group, tumors had disappeared or decreased in size in mice that survived for 60 days. The 2- and 4-week inoculation groups were more likely to have a longer survival than the 1-week inoculation group. Each treatment group had a significantly prolonged survival than the PBS group at 60 days (1 week, p = 0.001; 2 weeks, p < 0.001; and 4 weeks, p < 0.001) (Figure 7). There was a trend toward longer survival in the 4-week inoculation group than in the 1-week inoculation group (p = 0.0671). Therefore, the prolongation of survival with T-01 was dependent on the number of doses.

## Discussion

The T-01 used in this study can synthesize viral proteins only in proliferating cells due to the insertion of the LacZ gene within the ICP6 gene. However, it cannot synthesize proteins except in cancer cells due to the deletion of the γ34.5 gene. In addition, deletion of the α47 gene enhances viral replication exclusively in cancer cells and increases MHC class I expression in tumor cells, leading to enhanced tumor immunity. This genetically modified oncolytic virus infects and replicates within cancer cells, thereby exerting a cell-killing effect. The progeny of T-01 that replicate in cancer cells transfer from cell to cell and exert an antitumor effect via repeated cycles of replication, cell death, and infection. A critical feature of this oncolytic virus is that it does not replicate in, or harm, infected normal cells. T-01 has almost the same genome structure as G47Δ.^29^ In June 2021, G47Δ was approved under the generic name of teserpaturev after a phase II investigator-initiated clinical trial in patients with glioblastoma conducted in Japan.

The current study showed that oncolytic viral therapy provides an alternative treatment to chemotherapy for refractory sarcoma. Previous studies by Cripe and coworkers have found that the therapeutic effect of oHSV in immunocompetent sarcoma mouse models varied widely and that it was mediated by T cells.^32^^,^^33^ In addition, transforming growth factor-β inhibition could complement oncolytic herpes virotherapy of RMS by promoting an improved antitumor immunological response.^34^ Furthermore, the combination of virotherapy and immune checkpoint therapy was found to be effective against sarcoma.^33^ Therefore, third-generation genetically modified T-01 may have antitumor effects against advanced-staged, recurrent, or distant metastatic sarcoma. In our study, T-01 had good cytotoxic effects and replication capacity in RMS and LMS *in vitro* (Figures 1 and 2). Our *in vivo* experiments showed that inoculation with T-01 was effective in inhibiting tumor growth in the subcutaneous tumor models of LMS and RMS (Figure 3). Tumor suppression was inhibited by higher concentrations and more frequent administration of T-01. In these experiments, T-01 had a tumor-suppressive effect on LMS and RMS. Thus, T-01 may effectively suppress different types of sarcomas. The killing activity of T-01 against RKN was not correlated with the volume of virus (PFU). In addition to the replication level of T-01, we cannot deny the possibility that the efficiency of T-01 entry into cells and the expression level of HSV receptors in the cell lines used are involved in the cytotoxic activity of T-01.^35^, ^36^, ^37^, ^38^, ^39^, ^40^ Cripe and coworkers have revealed that human RMS cell lines expressed high levels of Nectin-1 and low levels of other HSV entry receptors, nectin-2, and herpesvirus entry mediator, compared with other pediatric tumors. Moreover, they assessed infected RMS cell lines with K26GFP (wild-type gD) or its receptor-restricted derivatives d5-28V (nectin-1 restricted) and A3C/Y38C (HVEM restricted). In all cases, the transduction efficiency of nectin-1-restricted viruses was comparable to that of wild-type HSV-1. Meanwhile, minimal transduction was observed with HVEM-restricted viruses. Thus, human and mouse RMS cell lines are susceptible to HSV-mediated gene transfer, and they primarily use the nectin-1 receptor for virus entry.^32^

Oncolytic viral therapy using T-01 destroys tumor cells via direct killing activity while preserving normal cells, recognizing the destroyed tumor cells as non-self cancer antigens, and enhancing the antitumor effect via T-cell-mediated tumor immunity. In immune-normal cases, the T-cell-mediated immune response is enhanced by T-01, thereby resulting in a stronger antitumor effect.^29^ In immune-normal mice, the virus was undetectable in distantly located tumors that were not inoculated with oHSV-1.^24^ However, tumor cells infected with HSV-1 lacking the α47 gene have increased the expression of MHC class I molecules and stimulated tumor immune cells to exert antitumor effects on distant tumors, compared with tumor cells infected with HSV-1 with residual α47 gene.^24^ Our previous study showed that T-01 treatment inhibited tumor growth on both the non-inoculated and inoculated sides compared with PBS treatment in immune-normal mice implanted with Hepa1-6 on the bilateral dorsal surfaces.^30^ The CD8+ splenocytes of mice in the T-01-treated group stimulated with hepa1-6 released higher levels of IFN-γ and exhibited a remarkedly increased proportion of lymphocytes that specifically recognize hepa1-6 tumor cells.^30^ In this study, we generated subcutaneous tumors and peritoneal dissemination metastatic tumors derived from CCRF S-180II cells, a mouse sarcoma cell line, in immune-normal mice. If the bilateral subcutaneous tumor models were inoculated with T-01 on one side only, the growth of tumors on both the non-inoculated and inoculated sides was suppressed compared with tumors in the PBS group (Figure 5). Furthermore, the splenocytes from T-01-treated mice showed increased secretion of the immune-enhancing cytokines INF-γ, IL-2, and IL-4 and decreased secretion of tumor-suppressive IL-10. Therefore, the proportion of tumor-specific lymphocytes increased in the immune-stimulated splenocytes (Figure 6). Th1 cells involved in the response to antitumor immunity produce IFN-γ and IL-2, whereas Th-2 cells produce IL-4.^41^^,^^42^ In addition, IL-10 contributes to cancer progression by downregulating MHC class II expression in antigen-presenting cells and MHC class I expression in tumor cells, thereby creating an immune-permissive environment.^43^ The increased level of cytokines that activate T cells is attributed to the increased proportions of CD4+ and CD8+ T cells. Immunohistochemical analysis revealed that the proportion of CD4+ and CD8+ lymphocytes was more likely to increase in tumors on both the inoculated and non-inoculated sides of mice in the T-01 group compared with the PBS group (Figure S1). The arming of oHSVs with transgenes is a useful strategy for adding specific antitumor functions to oncolytic viruses.^16^ T-01 is the base oHSV for arming interleukin-2, IL-18, soluble B7-1, or thrombospondin 1.^16^^,^^29^ Arming IL-12, IL-18, or soluble B7-1 oHSVs may significantly enhance antitumor efficacy via the enhanced induction of antitumor immunity. Combined with the systemic administration of immune checkpoint inhibitors is a reasonable strategy to enhance the efficacy of tumor lytic viruses.^16^ Current studies have confirmed the antitumor efficacy of T-01 when used as an armed base oHSV. However, the efficacy of armed oHSV with immune checkpoint inhibitors is still being investigated.

We assessed the antitumor effect of T-01 in a peritoneal dissemination model in immunocompetent mice implanted with CCRF S-180II cells (Figure 7). This model exhibited peritoneal dissemination nodules and massive bloody ascites. The PBS group had rapid tumor progression and death. Meanwhile, the T-01 group had a significant survival. In all patients (n = 10) in the 4-week T-01 treatment group, peritoneal nodules and ascites disappeared from the peritoneal cavity of the mice. Therefore, T-01 can selectively infect peritoneal disseminated nodules via ascites and can exert its cell-killing effect. Therefore, T-01 may be a fundamental treatment for different types of cancers that have progressed to peritoneal dissemination. The mechanism of the T lymphocyte-dependent response by T-01 is a puzzling issue, which deserves dedicated future studies.

In conclusion, T-01 can effectively inhibit tumor growth in the mouse models of RMS and LMS. Oncolytic viral therapy using third-generation oHSV may be a novel treatment for refractory sarcoma.

## Materials and methods

### Cell lines and viruses

The human RMS cell line RD, human LMS cell lines RKN and SKN, and mouse sarcoma cell line CCRF S-180II, which was derived from the ascites fluid, were purchased from the Japanese Collection of Research BioResources (Osaka, Japan).^44^, ^45^, ^46^ The human LMS cell lines SK-UT-1 and SK-LMS-1 were purchased from the American Type Culture Collection. The African green monkey kidney cell line vero and the human RMS cell line RMS-YM were obtained from RIKEN BioResource Center (Tsukuba, Japan).^47^^,^^48^ Viral stocks were prepared via high-speed centrifugation after the release of virus-infected vero cells using heparin, as described in a previous study.^29^

### Mice

Four-week-old male ICR mice and female athymic mice (BALB/c nu/nu) were purchased from Charles River Laboratories Japan, Inc. (Kanagawa, Japan) and were used at 5 weeks of age. The mice were caged in groups of four or fewer. Animal husbandry and experiments were performed according to the ARRIVE and PREPARE guidelines.^49^^,^^50^ Mouse experiments were conducted in accordance with the guidelines approved by the Animal Experiment Committee of Kansai Medical University (approval no. 20-048, 21-044).

### *In vitro* cytotoxicity and virus yield

*In vitro* cytotoxicity assays were performed, as described in previous studies.^24^^,^^51^ Cells were seeded in six-well plates, incubated at 37°C overnight, and then inoculated with virus or PBS for 1 h. The medium was removed, and the cells were incubated in fresh medium supplemented with 1% fetal calf serum at 34.5°C. The number of viable cells was counted for 4 days using the Coulter counter (Beckman Coulter, Fullerton, CA) and was expressed as percentage of the mock-infected control. To determine the virus yield, cells were seeded in six-well plates (5 × 10^5^/well) and cultured at 37°C for 24 h. The cells were then infected with T-01 at an MOI of 0.01 and were incubated at 37°C for 48 h. The titer of the progeny virus strain was assessed using plaque assay using vero cells. Each experiment was performed three times.

### Experiments using subcutaneous tumor models

Tumor cells (5 × 10^6^) were administered subcutaneously into the right flank of athymic mice (SK-LMS-1 and RMS-YM cells) or the bilateral flanks of ICR mice (CCRF S-180II cells). When the tumors reached a diameter of 5 to 7 mm, the animals were randomly assigned to the T-01 and PBS groups. T-01 virus (2.0 × 10^6^ PFU) was diluted to 20 μL in PBS containing 10% glycerol and was administered into the tumors of mice in the T-01 group. Meanwhile, only PBS was administered to mice in the PBS group.

In the first experiment, PBS or T-01 was administered into mice on days 0 and 3 (with a total of two inoculations). The tumor growth of the two groups (n = 10 per group) was compared. Further, it was monitored twice a week for 4 weeks using calipers after virus inoculation using the following formula: tumor volume (0.5 × [major axis] × [minor axis]^2^). In the second experiment, the virus concentration was kept constant at 2.0 × 10^6^ PFU. The mice in the PBS and T-01 groups were inoculated with PBS or T-01 twice a week for 1, 2, or 4 weeks (with a total of two, four, and eight inoculations, respectively) (n = 8 per group). In the third experiment, ICR mice with established subcutaneous tumors (from CCRF S-180II cells) on both flanks were used to examine the efficacy of T-01 in immunocompetent mice. On days 0 and 3, only one side of the tumor was treated with PBS or T-01 (with a total of two inoculations), and the PBS and T-01 groups were compared (n = 8 per group).

Mice were euthanized when they became moribund (lethargic, supine or prone, restricted gait in response to rough breathing or stimulation) or when the maximum tumor diameter exceeded 20 mm. Next, they were anesthetized via the intraperitoneal administration of pentobarbital. Subcutaneous tumors were harvested, fixed in 10% formaldehyde, and then embedded in paraffin for histological analysis. This study was performed based on the guidelines of the NIH Office of Animal Care and Use.^52^

### Peritoneal metastasis in mice

Peritoneal metastatic tumors were induced via the intraperitoneal administration of 5 × 10^6^ CCRF S-180II cells into ICR mice. The formation of peritoneal dissemination with bloody ascites was observed in all mice after 6 days. At the time of peritoneal metastasis, the mice were randomized and inoculated intraperitoneally with T-01 (2.0 × 10^6^ PFU) in 100 μL PBS containing 10% glycerol twice a week (days 0 and 3) at various time points (1, 2, and 4 weeks) or with PBS at the same time points. The day of the initial administration was set as day 0, and the overall survival time was examined.

### Histochemical analysis

#### H&E staining

Mice were killed on day 7 after two administrations of 2.0 × 10^6^ PFU T-01 or PBS on days 0 and 3. Subcutaneous tumor tissues were embedded in 10% formalin, and 5-μm-thick sections were placed on silanized slides (Dako Cytomation, Glostrup, Denmark) and stained with H&E.

### 5-Bromo-4-chloro-3-indolyl-β-D-galactopyranoside staining and immunohistochemical analysis of HSV-1

Mice were killed on day 7 after the administration of 2.0 × 10^6^ PFU T-01 or PBS on days 0 and 3. The samples were snap frozen in isopentane cooled with dry ice. Cryostat sections, with a thickness of 10 μm, were prepared from each sample. Sections were fixed in 2% paraformaldehyde in PBS for 10 min, washed three times in PBS, and incubated with PBS containing 2 mM magnesium chloride, 0.01% sodium deoxycholate, and 0.02% Nonidet P-40 (NP-40) at 4°C for 10 min. Sections were further incubated with substrate solution (PBS containing 1 mg/mL 5-bromo-4-chlor o-3-indolyl-b-D-galactopyranoside [X-Gal], 5-mM potassium ferricyanide, 5-mM potassium ferrocyanide, 2-mM magnesium chloride, 0.01% sodium deoxycholate, and 0.02% NP-40) at 32°C for 3 h and then washed once with water and twice with PBS containing 2-mM EDTA. Sections were counterstained with hematoxylin before mounting.^51^

Sequential sections were cut and subjected to immunohistochemical analysis to detect HSV-1. The sections were treated to inhibit endogenous peroxidase activity and prevent nonspecific binding of the secondary antibody, incubated with a rabbit polyclonal anti-HSV-1 antibody (1:50,000) (Dako Cytomation), rinsed, and then incubated with an horseradish peroxidase-conjugated goat anti-rabbit immunoglobulin (Ig)G antibody (Nichirei Bioscience, Tokyo, Japan). A positive reaction was visualized as a brownish color using 3-3′-diaminobenzidine as the chromogenic substrate.^30^^,^^31^

### Analysis of CD4 and CD8 expression

Subcutaneous CCRF S-180II tumors were established on the bilateral flanks of ICR mice, and only one tumor was inoculated with PBS or T-01 (2.0 × 10^6^ PFU) twice a week (days 0 and 3). Mice were killed 10 days after virus or PBS inoculation, and subcutaneous tumor tissues and spleens were embedded in optimal cutting temperature compound and frozen in liquid nitrogen. Sections (with a thickness of 5 μm) were mounted on silanized slides (Dako Cytomation). Samples were incubated with a rat anti-CD4 antibody (diluted 1:5) or a rat anti-CD8 antibody (diluted 1:10) (BD Pharmingen, San Diego, CA), followed by incubation with donkey anti-rat IgG (Jackson ImmunoResearch Laboratories, West Grove, PA). A positive reaction was visualized as a brownish color using 3-3′-diaminobenzidine as the chromogenic substrate. Sections were then counterstained with hematoxylin. CD4+ and CD8+ cells were counted within randomly selected intensely stained fields using a light microscope. The mean numbers of CD4+ and CD8+ cells per mm^2^ were counted (n = 3 per group).

### Enzyme-linked immunospot assay

Subcutaneous CCRF S-180II tumors were established on the bilateral flanks of ICR mice, and only one of the tumors was inoculated with PBS or T-01 (2.0 × 10^6^) twice a week (days 0 and 3). On day 10, the treated mice were euthanized, and splenocytes were collected. Splenocytes were evaluated for T-01 immunogenicity using the enzyme-linked immunospot (ELISPOT) assay, which detects IFN-γ, IL-4, IL-2, and IL-10. The splenocytes (6.0 × 10^5^) were added to the plate, and target cells were added to the plate and placed in an incubator containing 5% CO_2_ for 24 h at 37°C. ImmunoSpot S6 Analyzer (Cellular Technology Limited, Cleveland, OH) was used to automatically count the number of spots.

### Flow cytometric analysis

Subcutaneous CCRF S-180II tumors were established on the bilateral flanks of ICR mice, and only one of the tumors was inoculated with PBS or T-01 (2.0 × 10^6^) twice a week (days 0 and 3). On day 10, the treated mice were euthanized, and splenocytes were collected. GentleMACS dissociator and gentleMACS C tubes (Miltenyi Biotec, Bergisch Gladbach, Germany) were used to prepare splenocytes according to the manufacturer’s protocol. Red blood cells were removed using lysis buffer (BD Biosciences, San Jose, CA). Splenocytes were stained with several antibodies (Table S1) and analyzed using the gating strategy (Figure S3). Flow cytometric analysis was performed using the FACSCanto II flow cytometer (BD Biosciences).

### Statistical analysis

Data were presented as means ± SE. *In vitro* data and *in vivo* tumor volume data were compared using the Student’s t test. Multiple comparisons were evaluated using the Tukey-Kramer test. Overall survival was evaluated using the Kaplan-Meier method and was compared using the log rank test. A p value of <0.05 was considered statistically significant. Statistical analysis was performed using R version 3.4.3 (R Foundation for Statistical Computing, Vienna, Austria) and JMP 14.0.0 (SAS Institute, Cary, NC).

## Supplemental information

Document S1. Figures S1–S3 and Table S1

Document S2. Article plus supplemental information
