# Supplementary material for: Efficacy of a third-generation oncolytic herpes simplex virus in refractory soft tissue sarcoma xenograft models
Source: Mol Ther Oncolytics. 2022 Apr 26;25:225–35. doi: 10.1016/j.omto.2022.04.010 (PMC9118137; doi:10.1016/j.omto.2022.04.010)
Supplement: Document S1. Figures S1–S3 and Table S1 [file mmc1.pdf]

OMTO, Volume *25*

Supplemental information

Efficacy of a third-generation oncolytic herpes simplex virus in refractory soft tissue sarcoma xenograft models

Masahiko Hatta, Masaki Kaibori, Hideyuki Matsushima, Terufumi Yoshida, Tadayoshi Okumura, Mikio Hayashi, Kengo Yoshii, Tomoki Todo, and Mitsugu Sekimoto

Supplementary Table 1.

Antibodies used in the flow cytometry analysis.

Description	Fluorochromes	Company
Anti-mouse CD3e	APC	BD Biosciences
IgG1, k (anti-TNP), isotype Control	APC	BD Biosciences
Anti-mouse CD4	PE	BD Biosciences
IgG2a, k, isotype Control	PE	BD Biosciences
Anti-mouse CD8a	BV421	BD Biosciences
IgG2a, k, isotype Control	BV421	BD Biosciences
Purified rat anti-mouse CD16/CD32		BD Biosciences
7-AAD staining solution		BD Biosciences

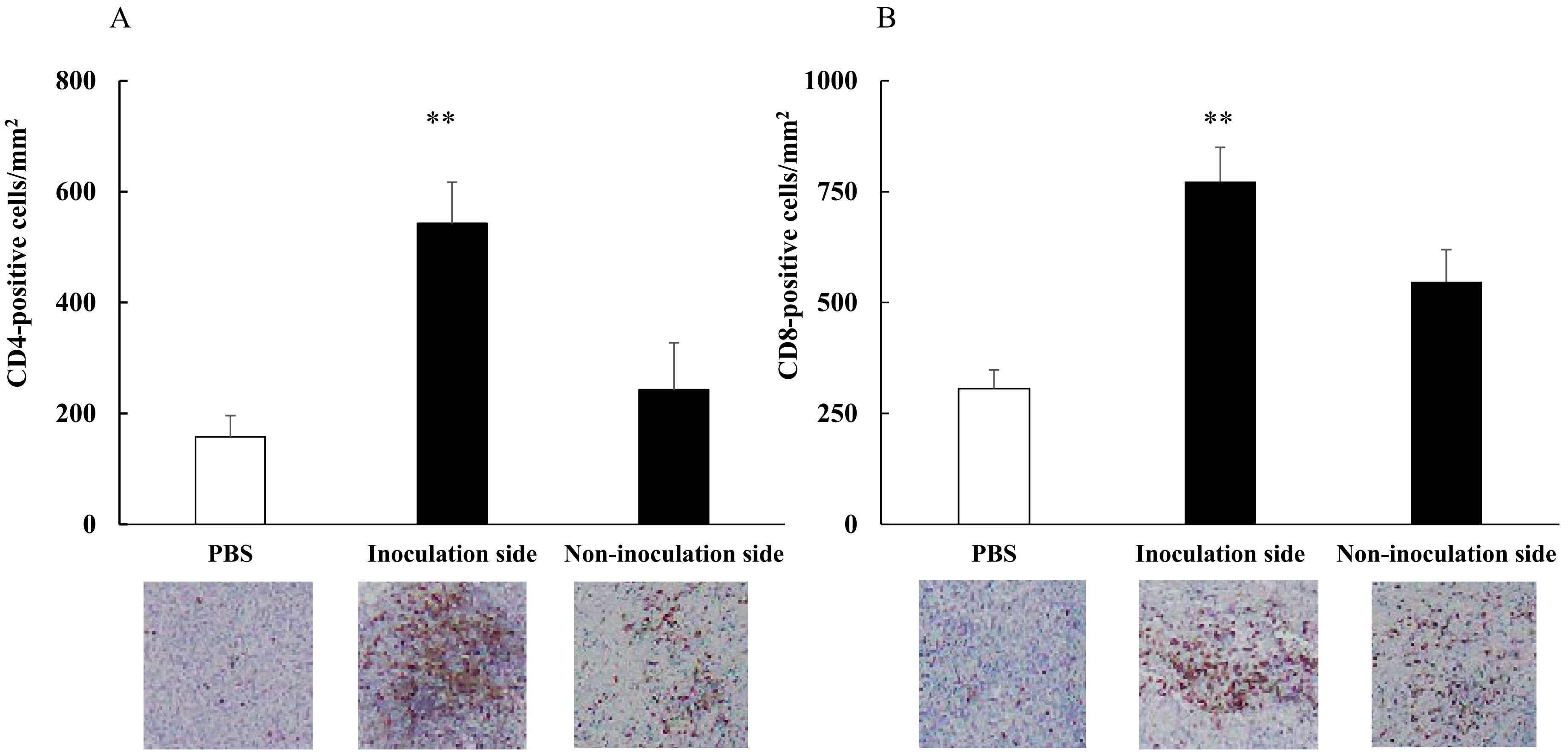

Supplementary Figure 1. Immunohistochemical analyses of CD8 and CD4

Male ICR mice with tumors established subcutaneously from CCRF S-180II cells on the bilateral dorsum were treated with PBS or T-01 (2.0×10^6 pfu) twice weekly (days 0 and 3). Sections prepared from tumors treated with T-01 (on the inoculation and non-inoculation sides) or PBS were immunostained using anti-CD4 (A) or anti-CD8 (B) antibodies. The graphs present the numbers of CD8+ or CD4+ cells/mm^2. Data represent the mean ± SE (n = 3 mice/group). Bar = 100 µm (magnification × 200). **P < 0.01 vs. PBS treatment.

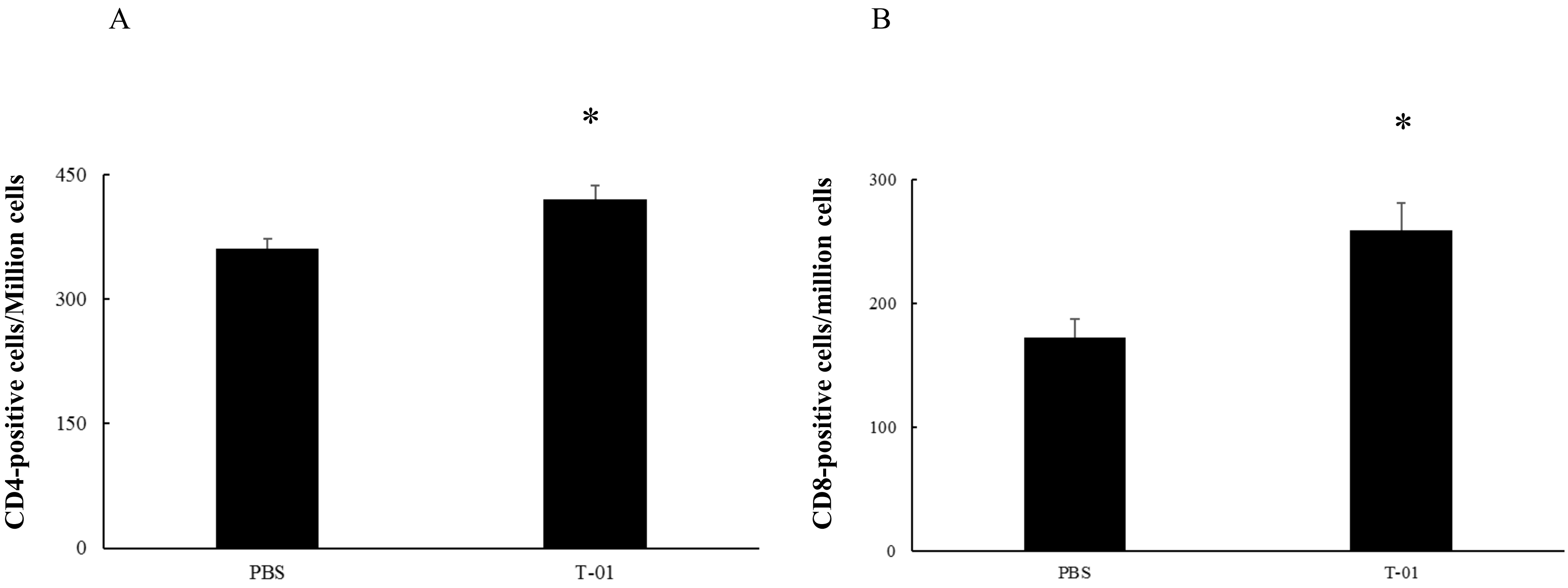

Supplementary Figure 2. Flow Cytometric Analyses of CD8 and CD4 cells

Male ICR mice with tumors established subcutaneously from CCRF S-180II cells on the bilateral dorsum were treated with PBS or T-01 (2.0×10^6 pfu) twice weekly (days 0 and 3). Sections prepared from tumors treated with T-01 (on the inoculation and non-inoculation sides) or PBS were immunostained using anti-CD4 (A) or anti-CD8 (B) antibodies. The graphs present the numbers of CD8+ or CD4+ cells/ million cells. Data represent the mean ± SE (n = 4 mice/group). *$P < 0.05$ vs. PBS treatment.

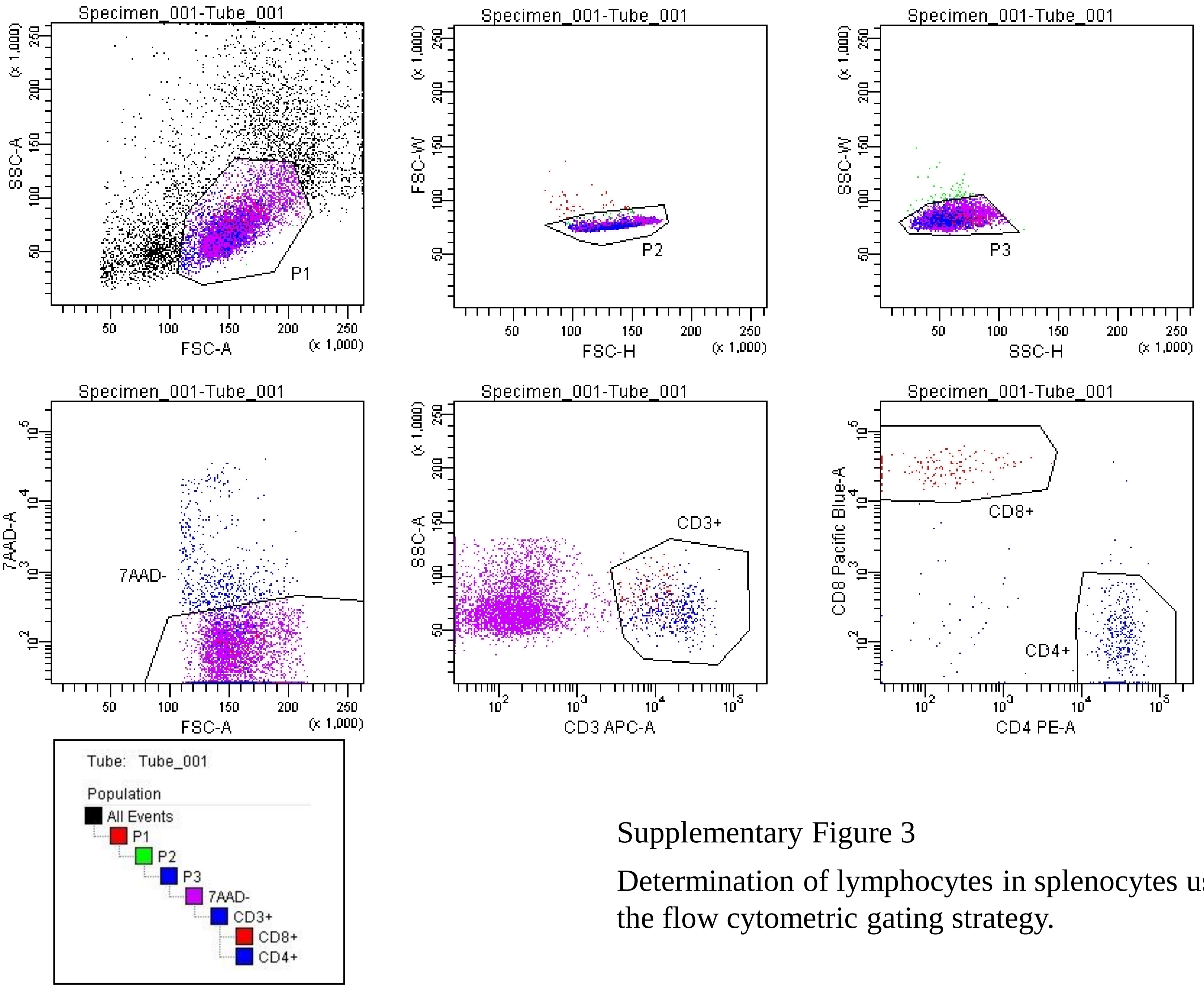

Supplementary Figure 3

Determination of lymphocytes in splenocytes using the flow cytometric gating strategy.
